# Differentiation of adipose-derived stem cells to chondrocytes using electrospraying

**DOI:** 10.1038/s41598-021-03824-5

**Published:** 2021-12-21

**Authors:** Nasim Nosoudi, Christoph Hart, Ian McKnight, Mehdi Esmaeilpour, Taher Ghomian, Amir Zadeh, Regan Raines, Jaime E. Ramirez Vick

**Affiliations:** 1 Biomedical Engineering Department, College of Engineering and Computer Science, Marshall University, Huntington, WV USA; 2 Mechanical Engineering Department, College of Engineering and Computer Science, Marshall University, Huntington, WV USA; 3 Computer Sciences and Electrical Engineering Department, College of Engineering and Computer Science, Marshall University, Huntington, WV USA; 4 Information Systems Department, College of Business, Wright State University, Dayton, OH USA; 5 Biomedical, Industrial and Human Factors Engineering Department, College of Engineering, Wright State University, Dayton, OH USA

**Keywords:** Cell biology, Materials science, Physics

## Abstract

An important challenge in the fabrication of tissue engineered constructs for regenerative medical applications is the development of processes capable of delivering cells and biomaterials to specific locations in a consistent manner. Electrospraying live cells has been introduced in recent years as a cell seeding method, but its effect on phenotype nor genotype has not been explored. A promising candidate for the cellular component of these constructs are human adipose-derived stem cells (hASCs), which are multipotent stem cells that can be differentiated into fat, bone, and cartilage cells. They can be easily and safely obtained from adipose tissue, regardless of the age and sex of the donor. Moreover, these cells can be maintained and expanded in culture for long periods of time without losing their differentiation capacity. In this study, hASCs directly incorporated into a polymer solution were electrosprayed, inducing differentiation into chondrocytes, without the addition of any exogenous factors. Multiple studies have demonstrated the effects of exposing hASCs to biomolecules—such as soluble growth factors, chemokines, and morphogens—to induce chondrogenesis. Transforming growth factors (e.g., TGF-β) and bone morphogenetic proteins are particularly known to play essential roles in the induction of chondrogenesis. Although growth factors have great therapeutic potential for cell-based cartilage regeneration, these growth factor-based therapies have presented several clinical complications, including high dose requirements, low half-life, protein instability, higher costs, and adverse effects in vivo. The present data suggests that electrospraying has great potential as hASCs-based therapy for cartilage regeneration.

## Introduction

Many scaffold fabrication techniques, such as gas foaming, fiber bonding, freeze drying, phase separation/inversion, and particulate leaching, provide little to no control over precise modification of structural characteristics of the scaffold^1^. While high precision techniques exist, such as bioprinting for the fabrication of 3D tissue structures via deposition of a bioink composed of cells and biomaterials^2^, none of these techniques can provide cellular stimulation in the way electrospraying can. This is an electrohydrodynamic process, like electrospinning, which involves the ejection of a stream of polymer solution or “melt” using an electrically charged jet, which can create fine droplets of varying size, depending on the strength of the electric field used. The combination of *polymers*, *electric field*, and *shear force* provided by electrospraying can be used as a means to provide cells with biophysical stimulation. These types of stimulation have previously been shown to induce differentiation in progenitor cells such as hASCs^3,4^. More specifically, biophysical cues like substrate properties and mechanical forces affect cell fate and differentiation. For example, an environment with high stiffness drives mesenchymal stem cells (MSCs) to undergo osteogenic differentiation, while low stiffness leads to lipogenic differentiation. Cyclic compression may cause MSCs to undergo chondrogenesis^5^. However, it is not known if these effects are independent of chemical/biochemical inducers such as growth factors. By focusing on biophysical cues independent of chemical/biochemical inducers, the specific mechanical determining factors of cell fate in vitro may be determined*,* as well as the physical elements that regulate and determine cell fate.

The classical electrospinning/electrospraying technique has been used successfully for nearly a century. Recently, however, a new form of cell electrospinning/electrospraying was developed to surmount the limitations of uneven cell distribution. Cell electrospinning/electrospraying produces fibers with living, viable cells encapsulated in hydrogels. This method allows the production of fibers with significantly higher resolution, improved ECM-like structure, and cell guidance along fiber channels, all with the inclusion of viable cells. However, the use of the aforementioned hydrogels as opposed to other polymers can significantly lower the mechanical strength of the structure as a whole while also maintaining some difficulty in controlling cell density^6^. One proposed method to counteract this deficiency in mechanical strength while also addressing the issues of harmful solvents and uneven cell distribution, is the use of core–shell technology, or electrospinning with a confocal needle that can accommodate for two different and separated polymer–solvent solutions during electrospinning^7^. Although past studies using this technique reported no loss in cell viability, none explored the effect on the cellular phenotype and differentiation capacity long after exposure to these strong electric fields^8–13^.

Many studies have shown the effect of low voltage electrical stimulation on chondrogenesis^14–16^. Endogenous electrical signals have been observed in articular cartilage during physiological processes, prompting the application of various types of electrical stimulation to in vitro chondrogenesis and in vivo cartilage repair^17^. Moreover, mechanical load is also one of several factors known to affect chondrogenesis of MSCs. One of the earliest events in response to mechanical load is an increase in intracellular Ca^2+^ levels. Several types of ion channels, including Transient receptor potential cation channel subfamily V member 4 (TRPV4), voltage operated calcium channels (VOCCs), and others have been recently demonstrated to play critical roles in controlling the intracellular Ca^2+^ responses of chondrocytes in the loaded cartilage^18^.

This study aims to introduce a new method to differentiate cells using electrospraying. This work is one of the first to study the specific effects of electrospraying on cell differentiation; it builds on prior work demonstrating that cells can survive electrospraying, and will advance a new approach of cell differentiation in tissue engineering.

## Materials and methods

### High-speed camera set up

The electrospraying setup consisted of a syringe pump (Fusion 101, Chemyx, Stafford, TX, USA), high-voltage power supply (HV350CC, Information Unlimited, Amherst, NH), two electrodes, a stainless steel needle, a syringe, and a collector plate. The syringe pump was used for controlling the volumetric flow rate of the solution. A capillary tube made from a stainless-steel needle (18G, Hamilton Company, Reno, NV, USA; inner diameter = 0.838 mm, outer diameter = 1.27 mm) was used to generate the electrospraying jet. The grounding plate was a 1.5 mm-thick aluminum plate with dimension of 60 mm × 60 mm. The steel needle was connected to a high-voltage electric source while the aluminum plate was grounded. The gap distance between the needle and aluminum collector was fixed at 70 mm. The flow rate was set as 200 μL/min, and the high voltages of 10 and 15 kV DC were applied to the nozzle. The high-speed camera (Phantom, VEO 440L, Amatek, Wayne, NJ)—integrated with a macro lens (12X Zoom lens with 12 mm fine focus, Navitar) and associated Phantom camera control software (PCC)—were used to capture the electrospraying mechanism. A continuous cold light was also used to capture jet images in high frequency. The high-speed camera was able to visualize and record the time-series images containing a complete periodic electrospraying cycle. The jet behavior and its initiation were recorded in a video format (512 × 1024 pixels at frame rate of 7000 fps). Each video was recorded for 5 s to capture 35,000 frames at each applied voltage, resulted in capturing the ejection of several droplets at each experiment.

### Electrical field simulation

The finite difference method (FDM) was used to determine the electric field distribution in the area of the device by solving Poisson’s equation. The code to solve this equation was written in MATLAB.

### Cell electrospraying

P2–P4 of adipose tissue-derived stem cells (hASCs) from ScienCell (Carlsbad, CA, USA) were used for cell cultures. Cells were plated in T75 culture-treated flasks with approximately 1 million cells per flask, and culture media was changed every 3–4 days for the duration of the culture. For the polymer solution, 125 mg gelatin and 125 mg pullulan were dissolved in 50 ml serum-free media. Media was warmed to 37 °C for gelatin dissolution. At the time of electrospinning, each mL of the Pullulan/Gelatin stock solution was mixed with another mL of serum-free media and was added to the cell pellet. Cell electrospraying content was aseptically transferred to a sterile 10 mL syringe, and a sterile 18-gauge syringe needle tip was secured. The collector plate, which was a Petri dish (Fisherbrand, polystyrene), was positioned 7 cm from the end of the needle tip. The syringe pump settings were adjusted to produce readings for a plastic 10 mL syringe pump. The pump volumetric flow rate was set to 200 μL/min. Electrospraying was performed at 10 and 15 kV. Control experimentation was performed without applying any voltages. Some electrosprayed samples were made using PKA-specific inhibitor H89 treated cells. The electrospraying setup is pictured in Supplementary Fig. S5.

### Viability test

The viability of the cells post-electrospraying was investigated by a live/dead assay kit and fluorescence microscopy. Approximately 6 h after electrospraying, the culture media was aspirated from each well. After incubation with calcein AM and ethidium homodimer (2 μM calcein and 4 μM ethidium in PBS) for 10 min at 37 °C, samples were washed with PBS and cells were imaged.

### Cytotoxicity test (lactate dehydrogenase (LDH) activity)

The media was aspirated, and cells were washed with PBS. Lactate dehydrogenase or LDH (Cytotox96 kit, Promega, Madison, WI, USA) was performed on the attached cells according to the manufacture’s protocol to look at the cell viability using cell lysate.

$${\text{Viability}}\% \, = \,{\text{Average}}\; {\text{OD}}\; {\text{of}}\; {\text{sample}}\, * \,{1}00{\text{/Average}} \;{\text{OD}}\; {\text{of}}\; {\text{control}}.$$

### Measuring spheroid size and area

Starting at 24 h after sample preparation, electrospun cells and control samples were imaged using the Keyence BZ-X810 fluorescent microscope (Itasca, Illinois) every day for two weeks as follows. Images were taken using the brightfield filter at 2.5 × zoom using a 2 × objective lens. Each image was taken with a 500 μm scale bar superimposed on the image. To ensure that images were representative of the dish as a whole, imaged regions were relatively randomly selected. Ten images for each dish were taken on each day for 14 days. The images were analyzed using the National Institutes of Health’s (NIH) ImageJ software. To analyze each image, the 500 μm scale bar was first used to set the scale of the image. Then, the image’s area was determined with the freehand measuring tool. Spheroids were considered as roughly spherical or rounded masses larger than approximately 2500–3000 μm^2^, with masses below 3000 μm^2^ considered only when found to have multiple cells gravitating around and oriented towards the mass, clearly indicating the formation of a spheroid rather than dead or floating cells/debris.

### Histology

Petri dishes of electrosprayed cells were taken after 4 and 7 days. After washing with PBS at room temperature, each well was fixed in 3.7% paraformaldehyde. For the chondrocyte proteoglycan examination, Petri dishes were stained with Alcian blue 8GX (Roth, Karlsruhe, Germany) and 0.01% (w/v) Safranin-O.

### Immunofluorescence

Cells were fixed with 3.7% paraformaldehyde, and permeabilized and blocked with 0.1% Triton X-100 triton and 0.5% bovine serum albumin. Cells were immunostained with anti-SOX9 (GMPR9; Thermofisher, Waltham, MA, USA), anti-aggrecan (Santa Cruz sc-33695) and Anti-Collagen II (NBP2-46,876; NovusBio, Littleton, CO, USA), antibodies, followed by DAPI (Life Technologies, Carlsbad, CA, USA). Cells were visualized with a Keyence BZX-810 microscope (Keyence, Osaka, Japan).

### Analysis of glycosaminoglycan (GAG) content

On days 14, and 21, the cell cultures were washed in PBS before being fixed using an acetone and methanol (1:1) solution at 4 °C for 3 min. One percent Alcian Blue in 3% acetic acid was added into the cell culture. The cells were incubated for 30 min and the overstaining dye was washed in 3% acetic acid and deionized water. One percent of Sodium dodecyl sulfate (SDS) was added to the cell culture and homogenized using a shaker at 200 rpm for 30 min. The absorbance was read using a microplate reader at 605 nm wavelength. The observation was repeated three times.

### ELISA

The cells were washed with PBS and lysed in lysis buffer with proteinase and phosphatase inhibitors. Protein concentrations were determined using the BCA Protein Assay Kit. The protein expressions of SOX9, Aggrecan and Collagen II were quantified using ELISA kit from MyBiosource Catalog No. MBS765509, MBS765509, and MBS765509 based on manufacturer protocol.

### Real time PCR analysis

Capacity cDNA Reverse Transcription Kit (Applied Biosystems, Foster City, CA, USA; Cat. no. 4368814) in a volume of 50 μL was used for real time PCR analysis. Primers for all assays were designed using Primer 3. Sequences for all assays are listed in Supplementary Table 1. Melting curve analysis was performed to ensure single-product amplification for all primer pairs.

Real time PCR was performed on the BioRad CFX384 Real Time System (BioRad, Hercules, CA, USA) using assays specific to the genes of interest. Each reaction well contained 5 μL of PowerUp™ SYBR Green Master Mix (Applied Biosystems, Foster City, CA, USA; Cat. no. A25742), cDNA equivalent to 20 ng of total RNA, and 250 nM each of forward and reverse amplification primers in a final reaction volume of 10 μL. Cycling conditions were as follows: 95 °C for 10 min for polymerase activation, followed by 40 cycles of 95 °C for 15 s and 60 °C for 1 min, with a final melting curve at the end of the thermal profile. Data analysis was performed using CFX Manager software from BioRad, version 3.1. The experimental Cq (cycle quantification) was calibrated against the endogenous control product glyceraldehyde-3-phosphate dehydrogenase (GAPDH). Samples were analyzed for relative gene expression by the $\mathrm{\Delta \Delta }Ct$ method^19^.

### Drawings

Figures were drawn with the help of biorender.com.

### Statistical analysis

In vitro experiments were performed *at least* in triplicate and repeated twice. The data was examined using MS Excel (Microsoft Corp., Redmond, WA, USA) and SAS JMP student edition 11 (SAS Institute, Cary, NC, USA). The quantified data from spheroid diameters, Alcian blue, gene expressions, and qPCR were compared using either unpaired two-tailed *t*-test, or one-way or two-way ANOVA. Post hoc comparisons (Tukey’s honestly significant difference) were used when significance was found. For RNA sequencing data, an exact permutation test was performed using the NPAR1WAY procedure in SAS. The data are expressed as the mean ± standard deviation; results were considered to be significant when *p* ≤ 0.05.

### RNA library preparation and sequencing

RNA sequencing libraries were prepared using TruSeq Stranded mRNA Library Prep Kit (Illumina, Inc., San Diego, CA, USA) according to the manufacturer`s protocol. The RNA concentration was measured with a Nanodrop 2000c spectrophotometer (Thermo Scientific Inc., Waltham, MA, USA). Integrity was assessed using Agilent 2200 Tapestation instrument (Agilent Technologies, Santa Clara, CA, USA). Briefly, polyA mRNA from an input of 500 ng high quality total RNA (RINe > 8) was purified and fragmented. First strand cDNA syntheses were performed at 25 °C for 10 min, 42 °C for 15 min and 70 °C for 15 min, using random hexameres and ProtoScript II Reverse Transcriptase (New England BioLabs Inc., Ipswich, MA, USA). In a second strand cDNA synthesis the RNA templates were removed, and a second replacement strand was generated by incorporation dUTP (in place of dTTP, to keep strand information) to generate ds cDNA. The blunt-ended cDNA was cleaned up from the second strand reaction mix with beads. The 3’ ends of the cDNA were then adenylated and followed by the ligation of indexing adaptors. PCR (15 cycles of 98 °C for 10 s, 60 °C for 30 s and 72 °C for 30 s) was used to selectively enrich those DNA fragments that have adapter molecules on both ends and to amplify the amount of DNA in the library. The libraries were quantified using the Promega QuantiFluor dsDNA System on a Quantus Fluorometer (Promega, Madison, WI). The size and purity of the libraries were analyzed using the High Sensitivity D1000 Screen Tape on an Agilent 2200 TapeStation instrument. The libraries were normalized, pooled, and subjected to cluster and pair read sequencing performed for 150 cycles on a HiSeqX10 instrument (Illumina, Inc. San Diego, CA, USA), according to the manufacturer's instructions.

### RNA-seq data analysis

Data was analyzed by ROSALIND® (https://rosalind.onramp.bio/), with a HyperScale architecture developed by ROSALIND, Inc. (San Diego, CA, USA). Reads were trimmed using cutadapt^20^. Quality scores were assessed using FastQC^21^. Reads were aligned to the *Homo sapiens* genome build hg19 using STAR^22^. Individual sample reads were quantified using HTseq^23^ and normalized via Relative Log Expression (RLE) using DESeq2 R library^24^. Read Distribution percentages, violin plots, identity heatmaps, and sample MDS plots were generated as part of the QC step using RSeQC^25^. DEseq2 was also used to calculate fold changes and p-values and perform optional covariate correction. Clustering of genes for the final heatmap of differentially expressed genes was done using the PAM (Partitioning Around Medoids) method using the fpc R library. Hypergeometric distribution was used to analyze the enrichment of pathways, gene ontology, domain structure, and other ontologies. The topGO R library^26^ was used to determine local similarities and dependencies between GO terms to perform Elim pruning correction. Several database sources were referenced for enrichment analysis, including Interpro^27^, NCBI^28^, MSigDB^29,30^, REACTOME, and WikiPathways^31^. Enrichment was calculated relative to a set of background genes relevant for the experiment.

## Results and discussion

**Figure 1:**
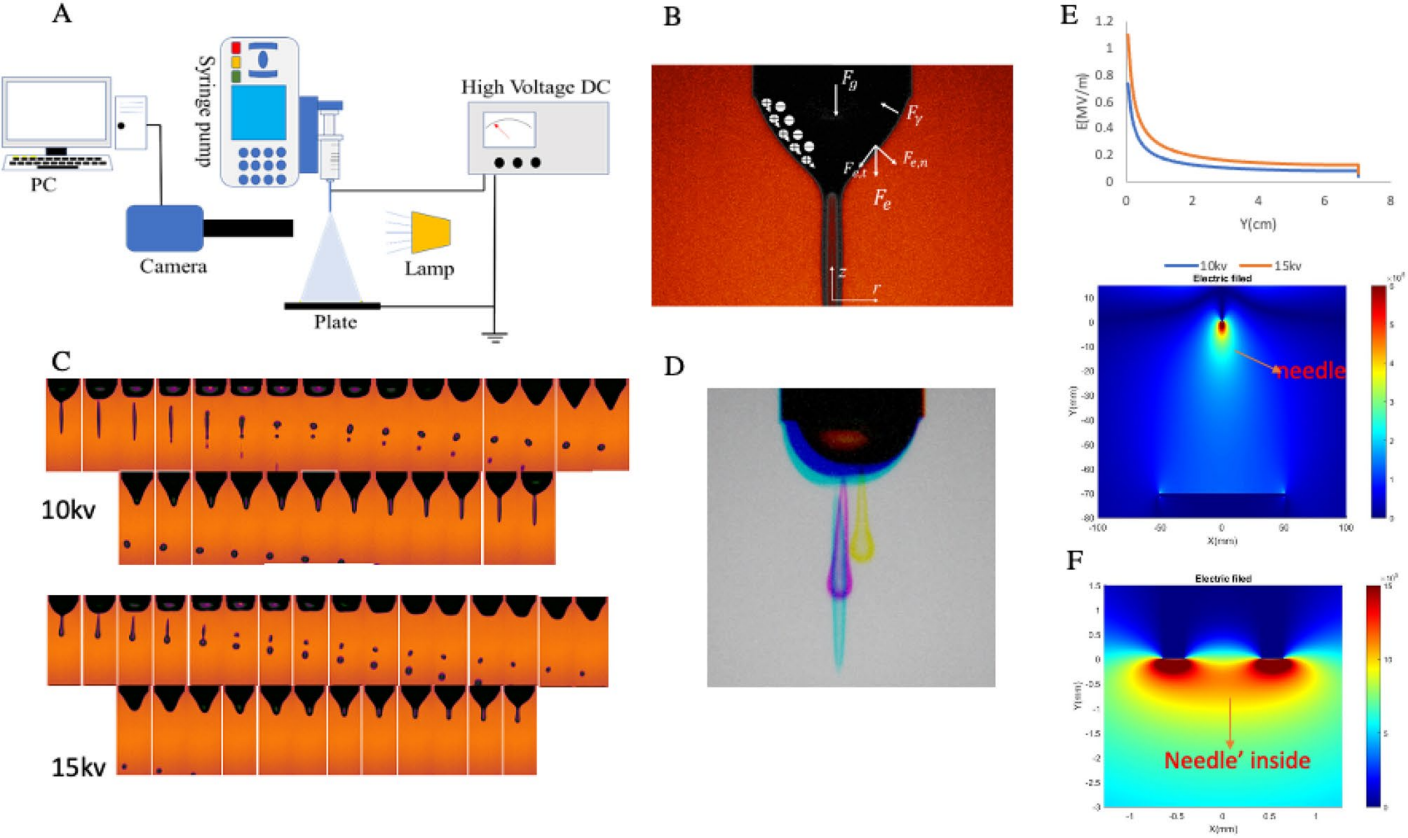
(**A**) Experimental setup. (**B**) Forces acting on the liquid during the formation of the cone-jet^35^. (**C**) The time series of different frames in micro-dripping mode at flow rate of 200 μL/min and electric voltage of 10 kV and 15 kV with $\Delta \mathrm{t}=142\mathrm{ \mu s}$. (**D**) The structure of jet cone at flow rate of 200 $\mathrm{\mu L}$/min and applied electric voltages of (yellow) 20 kV, (purple) 15 kV, and (green) 10 kV. (**E**) Electric field intensity on the Y axis connecting needle to ground plate. (**F**) Close look at the electric field distribution around and inside the needle.

In the experiment presented in Fig. 1A, the solution was supplied through the hypodermic needle connected to an adjustable high-voltage direct current (DC) power supply capable of providing a variable potential $\phi$. Under an applied electric field, the meniscus of diameter $D$ deforms into a droplet at the capillary tip. For a meniscus of diameter $D$ emerging from a charged capillary with internal diameter ${D}_{i}$ and external diameter ${D}_{o}$, the drop experiences several forces simultaneously: the electric force ${F}_{e}\sim {\varepsilon }_{0}{\phi }^{2}$ where ${\varepsilon }_{0}$ is the electrical permittivity of vacuum, the downward gravitational force is represented by ${F}_{g}\sim \rho {D}_{o}^{3}g$, the downward inertial force caused by the momentum per unit time injected by the flow into the meniscus is represented by $\dot{P}\sim \rho {Q}^{2}/{D}_{i}^{2}$, and the surface tension in the opposite direction is indicated by ${F}_{\gamma }\sim {D}_{o}\gamma$. Once the surface tension is overcome, fine droplets are formed. Due to their strong charge, these droplets are well-dispersed, diminishing the potential of agglomeration^32^. Electrospraying systems can operate under either dripping, micro-dripping, or oscillating micro-dripping modes depending on the flow rate and applied potential. Under our experimental flow rate and applied potential, the electrospraying system operated under the micro-dripping mode, due to the large electric force generated by the high applied potential, forming an elongated droplet shaped by the electric stresses. In this mode, the droplet size was much smaller than the outer diameter of capillary nozzle, in the range of 0.05${D}_{o}$- 0.85 ${D}_{o}$ (Fig. 1B)^33,34^.

Figure 1C shows the time series images, captured by a high-speed camera at flow rate of 200 $\mu \mathrm{L}$/min and applied electric voltages of 10 and 15 kV-DC, respectively. As a reference time for this sequence, the series starts with the first image showing the meniscus shape in which the detachment of the spindle-like fragment from the meniscus occurs. A stable electrospraying mechanism was observed with a steady flow of uniform non-aggregating microdroplets ejected downward. The microdroplets were usually polydisperse and with diameters in the range of 200–300 μm, with smaller outliers being less than 120 m$\mu$. Also, it was observed that a spindle structure formed in this micro-dripping mode in which the spindle-like fragment of liquid elongated by electrical forces was released from the tip of the capillary tube. The length and shape of this spindle-like fragment strongly depended on the applied electric voltage (see Fig. 1D). The detached spindle broke into different sized droplets which dispersed off the jet axis. As shown in Fig. 1C, for each applied voltage, the conical meniscus, following a periodic sequence of deformation to produce the droplets, displayed an axisymmetric shape with the tip of the cone on the jet axis, with droplets being ejected vertically toward the substrate. It is interesting to note that a small secondary daughter droplet was formed for both of the applied voltages tested. In the case of 10 kV DC, shortly after the spindle-like fragment of liquid leaves the meniscus, the stretching and conical breakup occured in the direction of the electric field, and the lobe formed at the tip of fragment moved off quickly away as a secondary daughter droplet. The formation of secondary droplet follows a different mechanism in the case of 15 kV DC. In this case, the secondary daughter droplet formed due to necking of the fluid into a filament which reshaped into the secondary daughter droplet between the meniscus and the mother droplet. Figure 1E shows the electric field intensity in the Y-axis connecting the center of the needle tip to the ground plate (this axis is shown in the lower figure) for both 10 kV and 15 kV. This figure illustrates that electric field intensity is high on the tip of the needle reaching 1.11 MV/m (0.74 MV/m) with an exponential decay to 129 kV/m (861 kV/m) near the ground plate for 15 kV (10 kV) applied potential. The electric field distribution around the needle tip provides some useful information. The inner and outer diameters of the needle were 838 and 1270 µm, respectively. The electric field distribution for the device with 15 kV applied potential, shown in Fig. 1F, illustrates that field intensity around the edge of the needle tip was extremely high and decreases as you move away. A close look at the electric field distribution around the needle tip, shown in Fig. 3F and Supplementary Fig. S1 reveals that cells are exposed to a nonuniform and ultra-high-intensity electric field while leaving the needle. The electric field on the X-axis on the needle tip for 15 kV applied potential was 804 kV/m at the center and almost double while moving toward the inner edge of the needle, to a level reaching 1.68 MV/m at less than 420 µm leading to a highly non-uniform electric filed around the tip. This unwanted intensive and nonuniform electric field may damage cells while leaving the syringe tip. Decreasing the applied potential voltage to 10 kV reveals that electric field intensity decreases by 33% reaching to 1.12 MV/m on the inner edge and 540 kV/m on the center of the needle tip.

**Figure 2:**
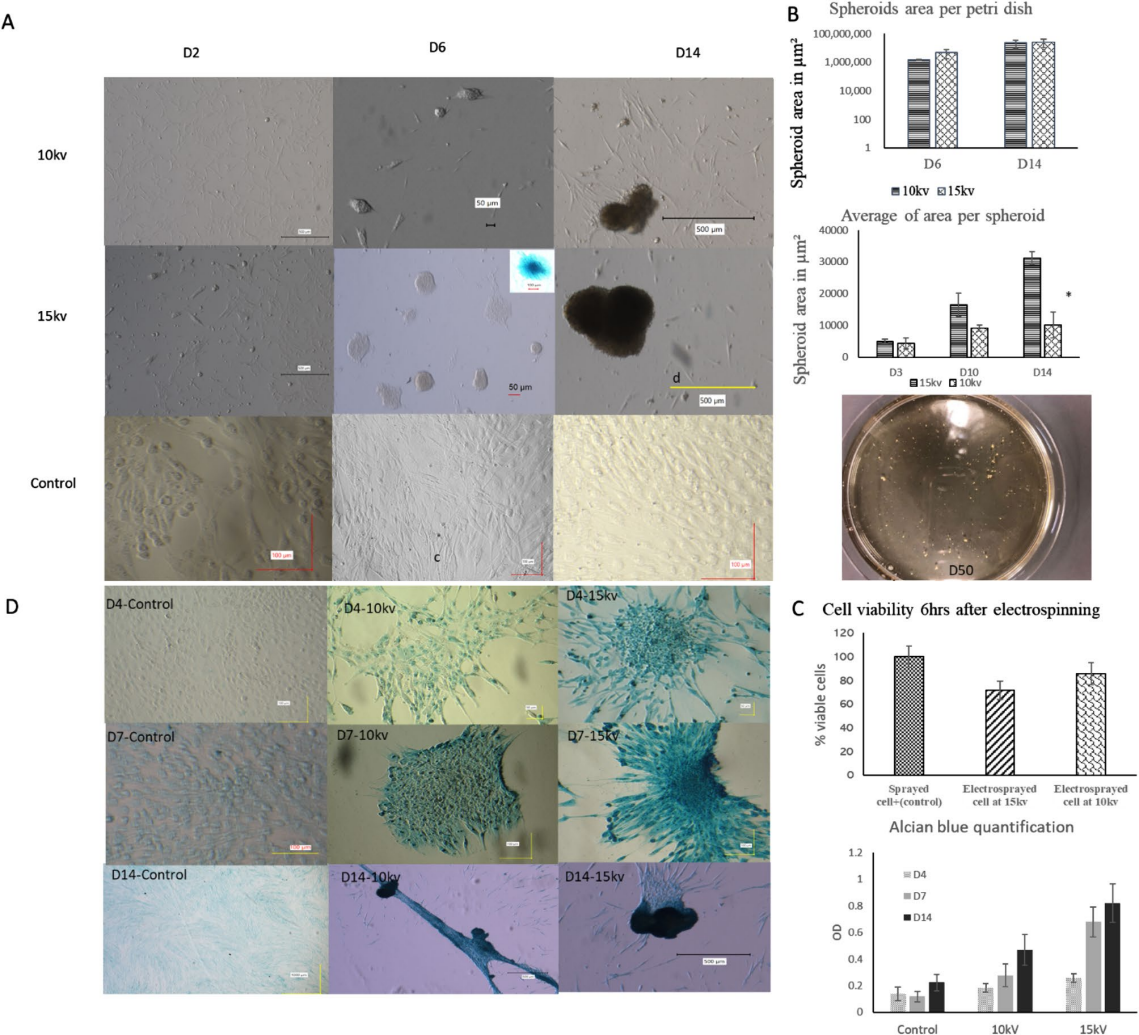
(**A**) Images of 10 kV, 15 kV, and negative control hASCs/gelatin/pullulan over time. On Day 2, no spheroids are visible. By Day 4, both the 10 and 15 kV dishes showed spheroids. On Day 14, spheroids were larger than at Day 6. Negative controls showed no spheroid formation. The positive control showing spheroid of chondrocytes is shown in Supplementary Fig. S3. (**B**, **C**) Comparison of spheroid area and cell viability at 10 and 15 kV. (**D**) (Upper) Alcian Blue staining of 10 kV, 15 kV, and control dishes after 4, 7, and 14 days, and their quantification. (*P* > 0.05, Tukey test). For surface measurement N = 5, and for Alcian blue and viability N = 3.

In the development of cartilage, the condensation of mesenchymal cells into spheroids—driven by TGF-β—marks the beginning of chondrogenesis^36^. By Day 4, spheroid (aggregate of cells) formation in both the 10 kV and 15 kV electrospun samples had begun, while no spheroid formation was observed in the control samples. This trend continued over time, with large multicellular spheroids becoming more common in both the 10 and 15 kV dishes past day 14, with no spheroid formation in the control samples (Fig. 2A). To quantify the spheroid growth, the area covered in a 2D microscopy image was measured. There was no statistically significant difference in the average total area of spheroids per Petri dish shown between the two electrospun groups at either Day 6 (*p* = 0.238) or Day 14 (*p* = 0.790), or from Day 6 to Day 14 in either the 10 kV (*p* = 0.133) or 15 kV group (*p* = 0.083). At Days 14, there was a significant increase in average area per spheroid in 15 kV group compared to the 10 kV group **however**, the total area of spheroids per petri dish by Day 14 was not significantly different between the two voltages (Fig. 2B).

Using standard electrospraying conditions at 10 kV, cell viability using Pullulan/Gelatin/hASC formulation was 90%, but at 15 kV using the same polymers, the viability was reduced to about 70% (Fig. 2C). The petri dishes were kept until day 50, and their viability was measured using Dead/live assay (Supplementary Fig. S2). Furthermore, we examined the effect of voltage on hASC differentiation. Production of glycosaminoglycans (GAGs) on the grown culture was analyzed based on Alcian Blue absorbance at 650 nm. Both the absorbance value of stained cells at 10 and 15 kV increased gradually from day 7 to 14. The absorbance value at 15 kV was higher than any other group, showing the maximum levels of chondrogenesis. Alcian blue staining images are shown in Fig. 2D.

**Figure 3:**
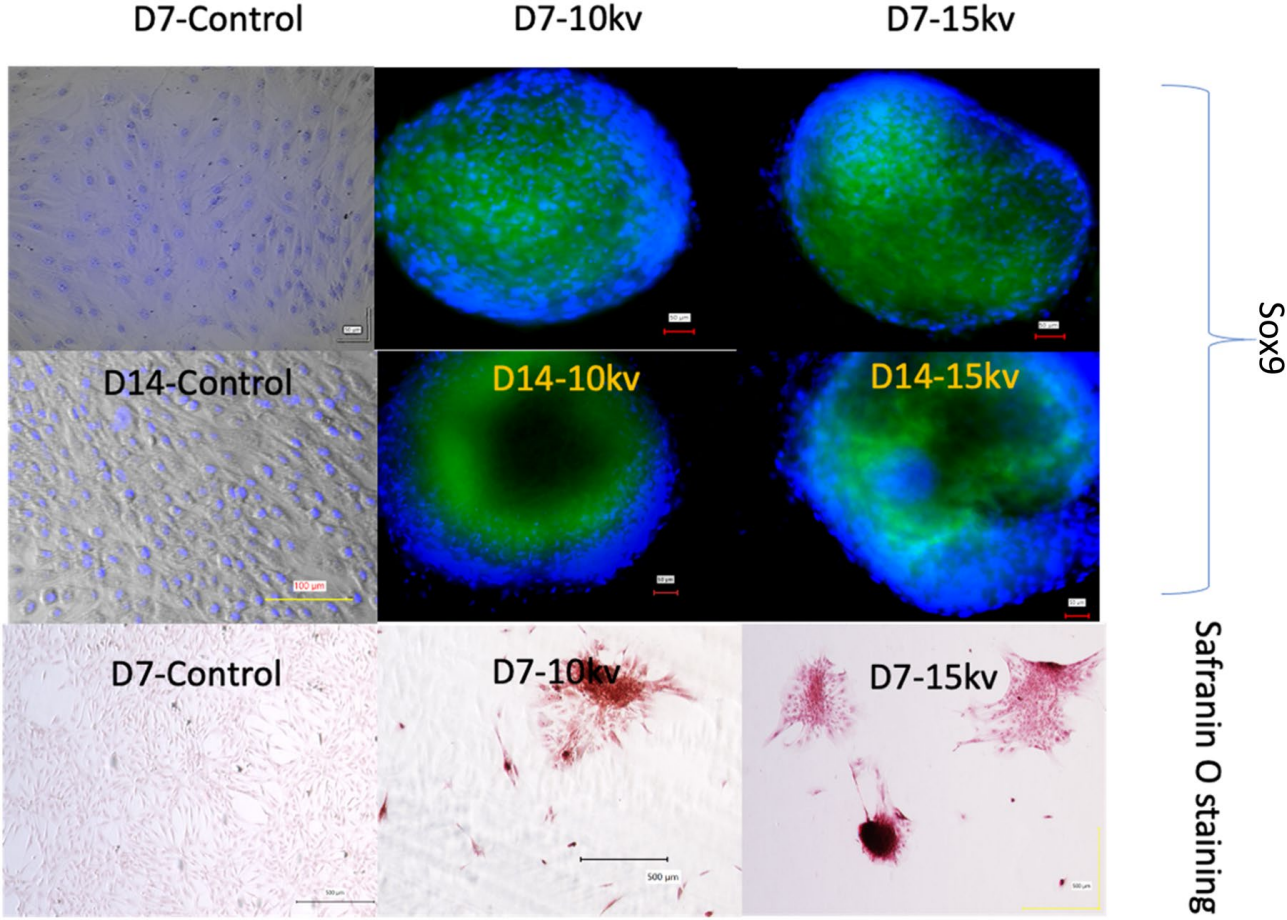
Sox9 and safranin O staining of 10 kV, 15 kV, and control of one single spheroid. The dishes were counterstained with DAPI (blue).

**Figure 4:**
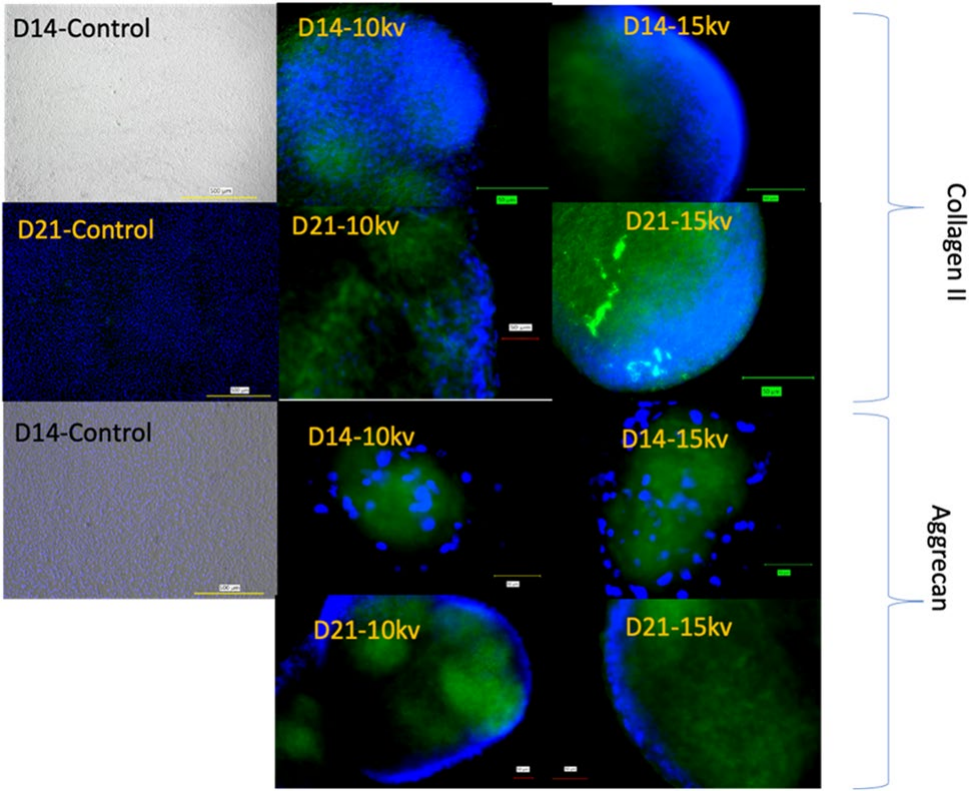
Collagen II and aggrecan (green) staining of 10 kV, 15 kV, and control of one single spheroid. The dishes were counterstained with DAPI (blue).

Expression of the transcription factor Sox9 induces condensed mesenchymal stem cells to differentiate into proliferating chondrocytes, marking an additional step in chondrogenesis^37^. Sox9 was observed at both 10 and 15 kV after 14 days and was present until day 21 (D21) (Fig. 3). The presence of Sox9 only within the electrospun samples supports the notion that electrospraying at 10 or 15 kV can successfully produce chondrocytes without the use of exogenous growth factors. Enzyme-linked immunoassay (ELISA) quantification showed that Sox9 showed significantly higher expression at 15 kV than at 10 kV at D14, but that there was no difference in expression by D21. Sox9 is required for chondrocyte proliferation and survival but delays prehypertrophy. The major upregulation of Sox9 revealed in this study under electrospraying conditions indicates the inhibition of osteoblastic differentiation. ^38^As seen in Fig. 3, by D7, Safranin O staining showed the presence of proteoglycans in both the 10 and 15 kV electrosprayed samples but not in the control samples. Similarly, collagen II and aggrecan (Fig. 4) were observed at both D14 and D21 in the 10 and 15 kV electrosprayed samples but not in the control samples. ELISA quantification confirmed that collagen II and aggrecan were expressed at D14 and D21 in electrosprayed samples, and were unaffected by the differences in voltage used in the electrosprayed samples (ELISA quantification is shown in Supplementary Fig. S4).

### Analysis of differentially expressed genes (DEG) during chondrocyte differentiation.

**Figure 5:**
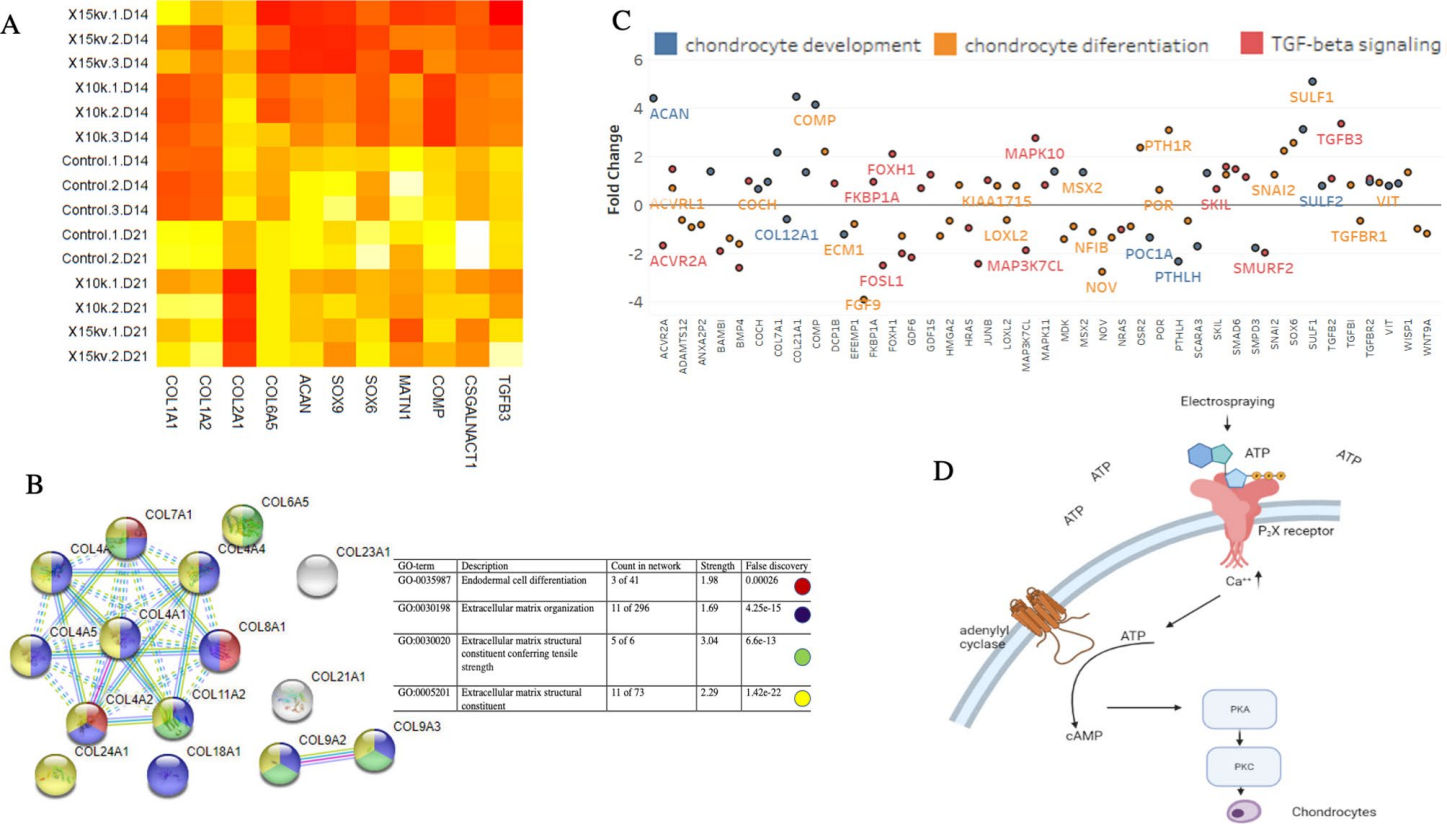
(**A**) Heat map of chondrogenesis related genes in control, 10 and 15 kV sample at D14 and D21 (N = 4). (**B**) Upregulated collagen genes network. (**C**) Enrichment analysis comparing electrosprayed and control samples. Y axis shows fold change. (**D**) Schematic of cell response to electrospraying.

To reveal the differentiation associated with phenotype change, a DEG analysis was performed to identify gene expression changes between electrosprayed and control samples. A total of 2257 genes (|fold change|> 1.5 and *p* < 0.05) were upregulated and 2140 genes were downregulated when comparing the 15 kV sample to the control. Once we compared the control to 10 kV sample, a total of 2012 genes (|fold change|> 1.5 and *p* < 0.05) were upregulated and 2208 genes were downregulated. Upregulated genes related to chondrogenic differentiation are shown in Fig. 5A. Aggrecan (*ACAN*) was upregulated at 15 kV (8.7-fold) and 10 kV (4.4-fold) at D14. Sox9 was upregulated sixfold at 15 kV and threefold at 10 kV at D14. These results were confirmed by PCR and are shown in the supplementary data. The expression of other extracellular matrix (ECM) genes like *COL2A1* has its maximum upregulation after 21 days. Many other collagen types were identified as upregulated (COL23A1, COL21A1, COL4A1, COL6A5, COL4A4, COL7A1, COL4A6, COL4A5, COL24A1, COL9A2, COL4A2, COL8A1, COL9A3, COL11A2, and COL18A1) or downregulated (COL17A1, COL19A1, COL13A1, COL15A1, COL8A2, and COL1A1). Therefore, we performed protein–protein interaction (PPI) network analyses using the search tool for retrieval of interacting genes (STRING) (https://string-db.org) database using the upregulated collagen genes.

Functional enrichments in this network showed that the ECM structural constituent conferring tensile strength has the highest strength (Fig. 5B). Moreover, SAA1, TEAD2, and KCTD12 were significantly higher in electrosprayed samples than the control after 14 days, as seen previously^39^*.* Genes at D14 were enriched for three pathways and biological process of TGF-β regulation, chondrocyte development, and differentiation (Fig. 5C). Of the 20 genes that are identified by the gene ontology term “CHONDROCYTE_DEVELOPMENT”, 17 genes were upregulated and 3 were downregulated. Looking at genes associated with the GO Term “CHONDROCYTE_DIFFERENTIATION” of the 56 genes, 30 genes were upregulated, and 26 genes were downregulated.

For “CHONDROCYTE_PROLIFERATION” 4 genes were upregulated, and 4 genes were downregulated. Genes such as ACAN, SULF1, SMAD3, SOX9, ZBTB16, COL21A1, COMP, PTH1R, SOX6, OSR2, SOX5, CYTL1, COL7A1, AXIN2, MATN1, WISP1, MSX, COL27A1, SFRP2, and SNAI2 were upregulated. Genes like NOV, INHBE, GDF6, and GDF5 were downregulated. Looking at the TGF-β signaling pathway, TGFB3, MAPK10, SMAD9, TGFB2, SMAD6, SMAD3, and JUNB were upregulated. The TGF-β signaling pathway has multiple biological functions and can regulate cell proliferation, differentiation, migration, and apoptosis^40^. The upregulation of 17 genes and downregulation of 11 genes seems to indicate that TGF-b signaling has an important role in electrospraying-stimulated chondrogenesis. Among BMPs, BMP6 was upregulated. An effect of direct electrical signal on protein kinase inhibitor A has been reported before^41^. To elucidate the mechanism behind the chondrogenesis in our experiment, cells were cultured with specific inhibitor H89 (20 µM) for 1 day and then electrospun at 10 and 15 kV. After 4 and 7 days, fewer than 5 visible aggregates were visible in the petri dishes.

## Discussion

There was no significant difference in gene expression for collagen II in the 10 and 15 kV samples between D14 and D21. Sox9 was significantly (*p* = 0.0005) higher in 15 kV at D14 compared to 10 kV, but not at D21. Similarly, Aggrecan was significantly higher at 15 kV in D14 compared to 10 kV but not at D21. Genes were also enriched for TGF-β regulation of ECM, focal adhesion, and E2F-mediated regulation of the DNA replication pathway. For this last effect, 12 genes including TYMS, POLA1, CDC25A, FBXO5, CDC45, ORC1, DHFR, ORC6, CDT1, CDC6, CCNB1, and RRM2 were downregulated. Non-canonical activities of E2Fs transcription factors with cell cycle regulators known as pocket proteins (PPs) have now been identified in stem and progenitor cells. It has been shown that E2Fs/PPs affect cell cycle regulation and cell fate decisions^42^. So, electrospraying might influence differentiation via E2F-mediated regulation of DNA. We have also observed that spheroids from 15 kV electrosprayed cells have the tendency to attach to each other more than those exposed to 10 kV. Chondrocytes have been reported to express several integrins, including those for type II and type VI collagen (α1β1, α2β1, and α21β1), vitronectin, laminin (α6β1), and most abundantly for fibronectin (α5β1). Interestingly, the upregulated genes at 15 kV compared to 10 kV, included ITGA4, ITGA6, ITGA7, ITGA8, and laminin subunit α 3.

Endogenous electrical signals have been observed in articular cartilage during physiological processes, prompting the application of various types of electrical stimulation for in vitro chondrogenesis and in vivo cartilage repair^17^. It has been shown that application of a 2 V/cm DC electric field can cause intracellular ATP depletion. However, the exact mechanism of intracellular ATP depletion and extracellular ATP increase in response to an electric stimulation is not clear. One possible cause may be a decrease in intracellular ATP due to transiently intensive ATP consumption by the cellular biomolecular machinery in response to DC field-mediated changes in the cell metabolism^43,44^. Another possible reason for intracellular ATP depletion and extracellular ATP increase could be that mechanical stress causes ATP release through hemichannels^45^. Our hypothesized mechanism by which electrospraying can induce chondrogenesis is as follows: we theorize that electrospraying can cause extracellular ATP increase. This extracellular ATP can bind and activate P2X4 receptors, leading to membrane depolarization by inducing the influx of cations such as Ca^2+^and Na^+^ and subsequently opening voltage-dependent calcium channels (VDCC). Finally, ATP releases via exocytosis by enhancing Ca^2+^influx (Fig. 5D). This positive feedback may initiate Ca^2+^ oscillations and subsequent ATP oscillations^46^ initiating chondrogenesis^17^.

Ca^2+^ oscillations drive the oscillations of (cyclic adenosine monophosphate) **cAMP**/(cAMP-dependent protein kinase A) and **PKA** signaling by controlling cAMP production. These cAMP oscillations arising from Ca^2+^ oscillations have been previously reported^47^. ATP is converted to cAMP by adenylyl cyclase (AC), and cAMP/PKA signaling regulates the oscillations of cAMP/PKA signaling. These facts suggest that cAMP/PKA signaling can mediate the coupling between Ca^2+^ and ATP oscillations in chondrogenesis. Therefore, Ca^2+^ oscillations can drive ATP oscillations, depending on cAMP/PKA signaling^46^. PKA signaling promotes chondrogenic differentiation by activating PKCα and enhancing SOX9 transcriptional activity^48^. PKCα mediates chondrogenesis via the ERK1/2 pathway^49^. Treatment for cartilage defects (e.g., articular hyaline cartilage defects) is a challenge, and the use of mesenchymal stem cells (MSCs) offers a potential alternative for cartilage engineering, since they can differentiate into chondrocytes in vitro. The process of chondrogenic differentiation of MSCs is commonly performed with a pellet or aggregate culture system, inducing differentiation through the addition of TGF-β, BMP and/or IGF^50^. Chondrocytes generated from MSCs express classical genes/proteins as native chondrocytes, e.g., type II collagen and aggrecan. However, it is also possible to identify hypertrophy-associated genes, as type X collagen, ALP and MMPs.

## Conclusion

Chondrocytes can be used in the treatment of focal cartilage injuries to prevent the onset of osteoarthritis (OA), and currently autologous chondrocytes are being used. This work suggests to generate chondrocytes from hASCs regardless of the limited capacity of autologous chondrocytes to expand. Electrical stimulation also shows great potential to differentiate stem cells so the results from this study suggest that this technique can be an alternative method for differentiating hASCs into chondrocytes—and ultimately usable cartilage—for regenerative medicine applications.

## Limitations of the study

Our hypothesis, based on preliminary results, is that both electrical stimulation and mechanical forces are needed to differentiate stem cell into chondrocytes. This is the biggest limitation of the current study.

In future studies, we aim to characterize cell differentiation in two different settings: (1) when cells are only stimulated by the voltage mimicking the electrospraying electric field and (2) when cells are solely stimulated by shear stress, mimicking electrospraying mechanical forces.

Moreover, in the current electrospraying setup, there is a non-uniform electric field. Exposing cells to the highest electric field during the electrospraying process might cause cell death, as was reported in the 15 kV samples previously. We need to improve our setup with a more evenly distributed electric field for future studies.

## Supplementary Information

Supplementary Figures.

## Data Availability

The datasets generated during and/or analyzed during the current study are available from the corresponding author on reasonable request.
