# Supplementary material for: Differentiation of adipose-derived stem cells to chondrocytes using electrospraying
Source: Sci Rep. 2021 Dec 21;11:24301. doi: 10.1038/s41598-021-03824-5 (PMC8692477; doi:10.1038/s41598-021-03824-5)
Supplement: Supplementary file 1 — Supplementary Figures. [file 41598_2021_3824_MOESM1_ESM.docx]

## ***Supplementary figures***


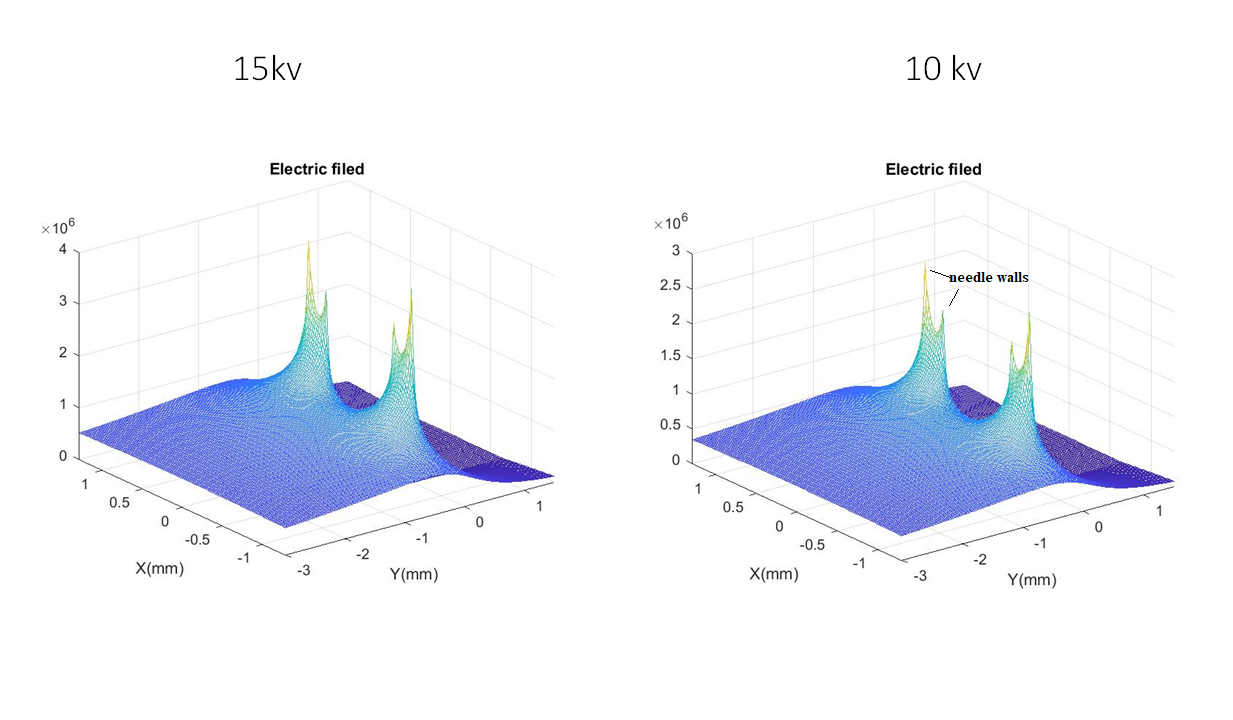


**Supplementary figure S1**: Electrical field is maximum at the inner and outer points of the needle.


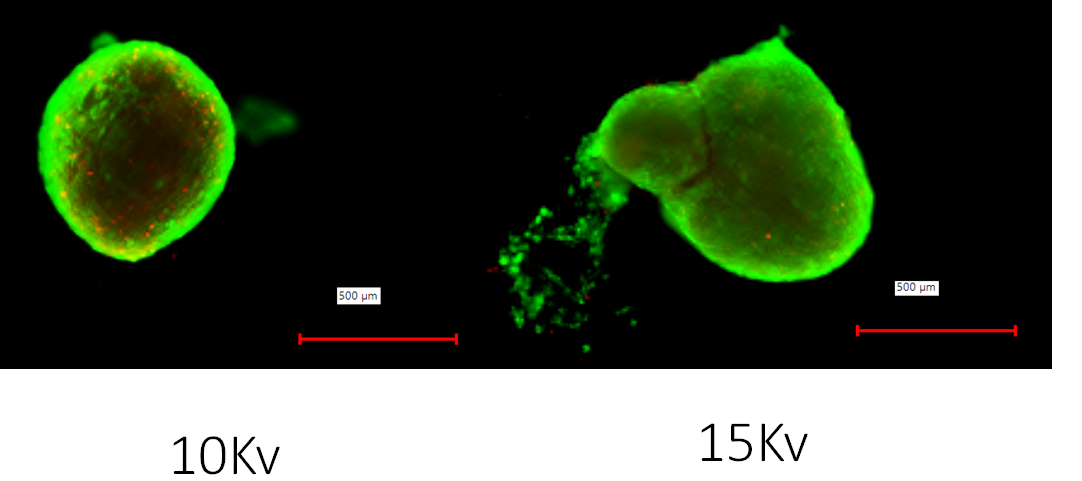


**Supplementary figure S2**: LIVE/Dead after 50 days of culture

*
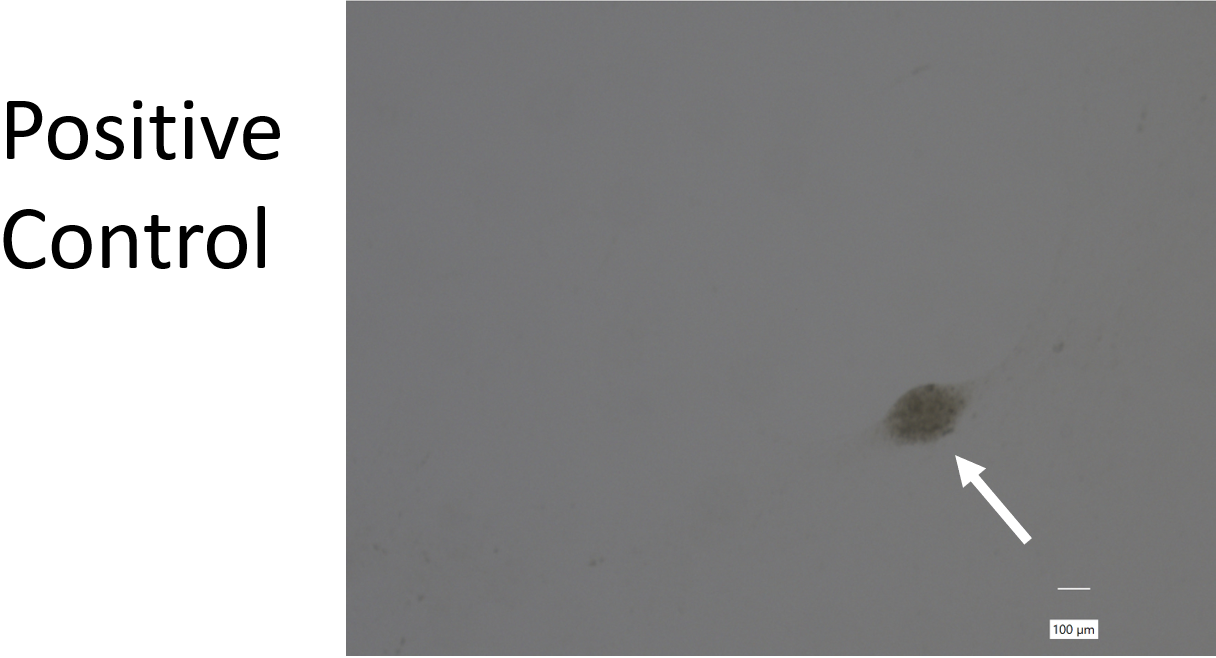
*

**Supplementary figure S3**: spheroid of chondrocytes

**Supplementary figure S4**::ELISA quantification. N=4

**Supplementary figure S5**: mRNA relative expression


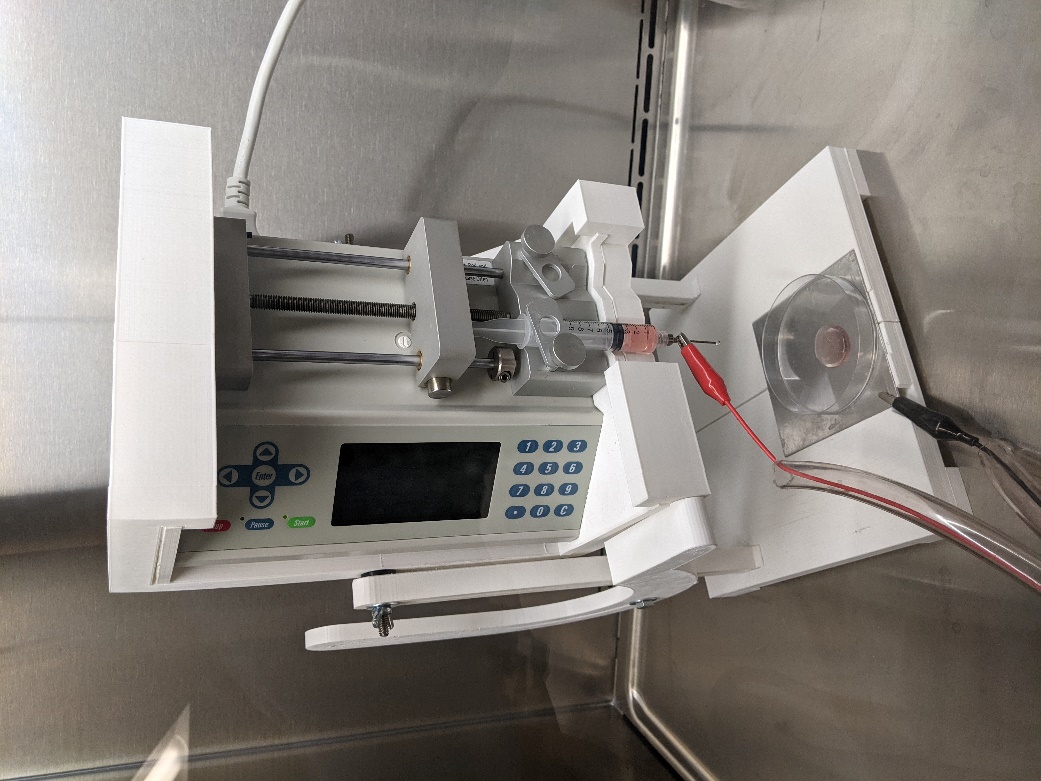


**Supplementary figure S6**: Electrospraying setup

**Supplementary table S1**: Sequence of Primer used.

| ***SOX9*** | ***Sequence (5'->3')*** |
| --- | --- |
| ***Forward primer*** | *GGCAAGCTCTGGAGACTTCTG* |
| ***Reverse primer*** | *CCCGTTCTTCACCGACTTCC* |
| ***Product length*** | *138* |
| ***ACAN*** | ***Sequence (5'->3')*** |
| ***Forward primer*** | *GCGAGCACTGTAACATAGACAT* |
| ***Reverse primer*** | *TCAATCTCACACAGGTCCCC* |
| ***Product length*** | *133* |

*
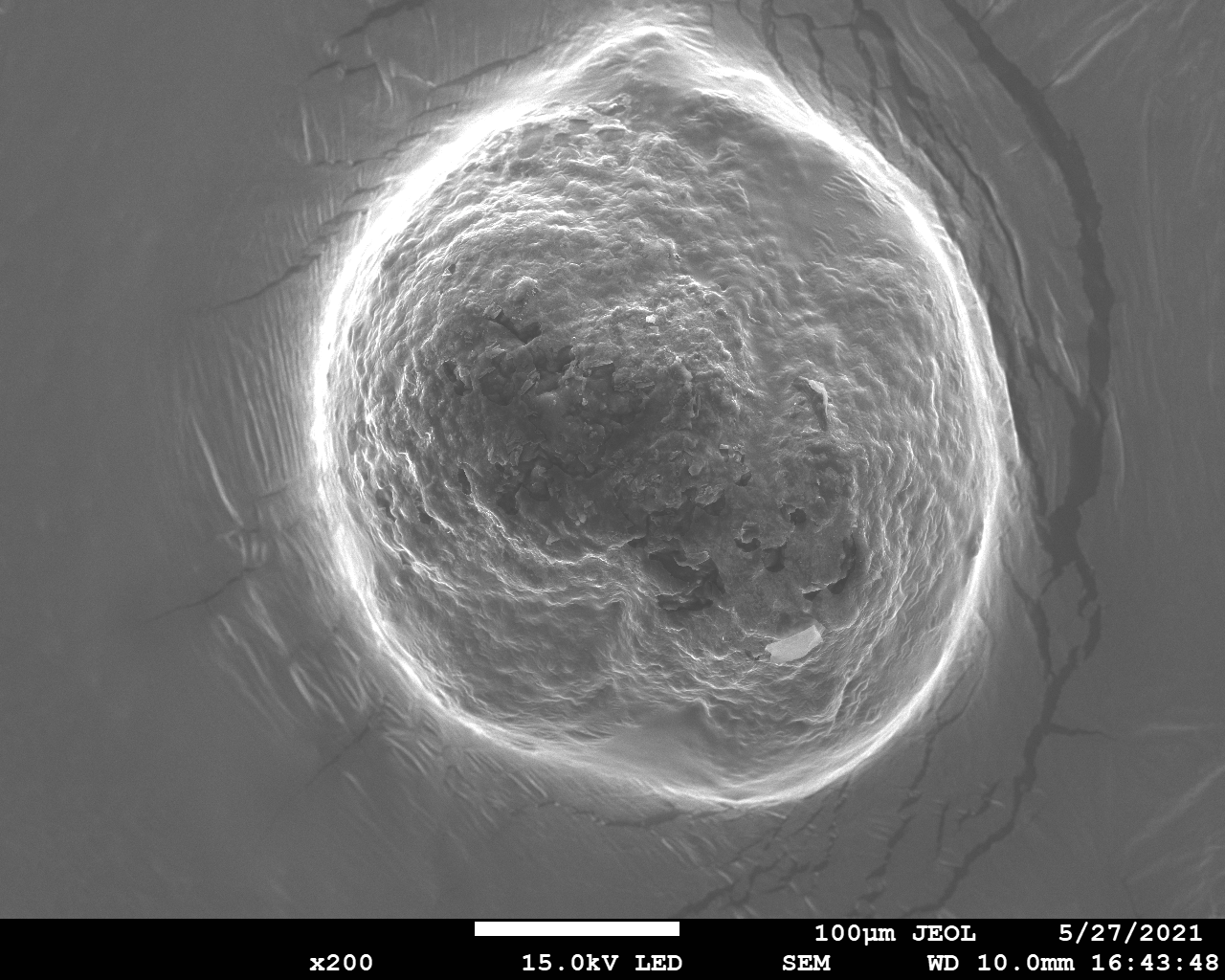
*

**Supplementary figure S7**: Scanning electron Microscopy of a spheroid of 15kv group at D21

**Supplementary figure S8**: Proliferation behavior of cells after electrospinning

*
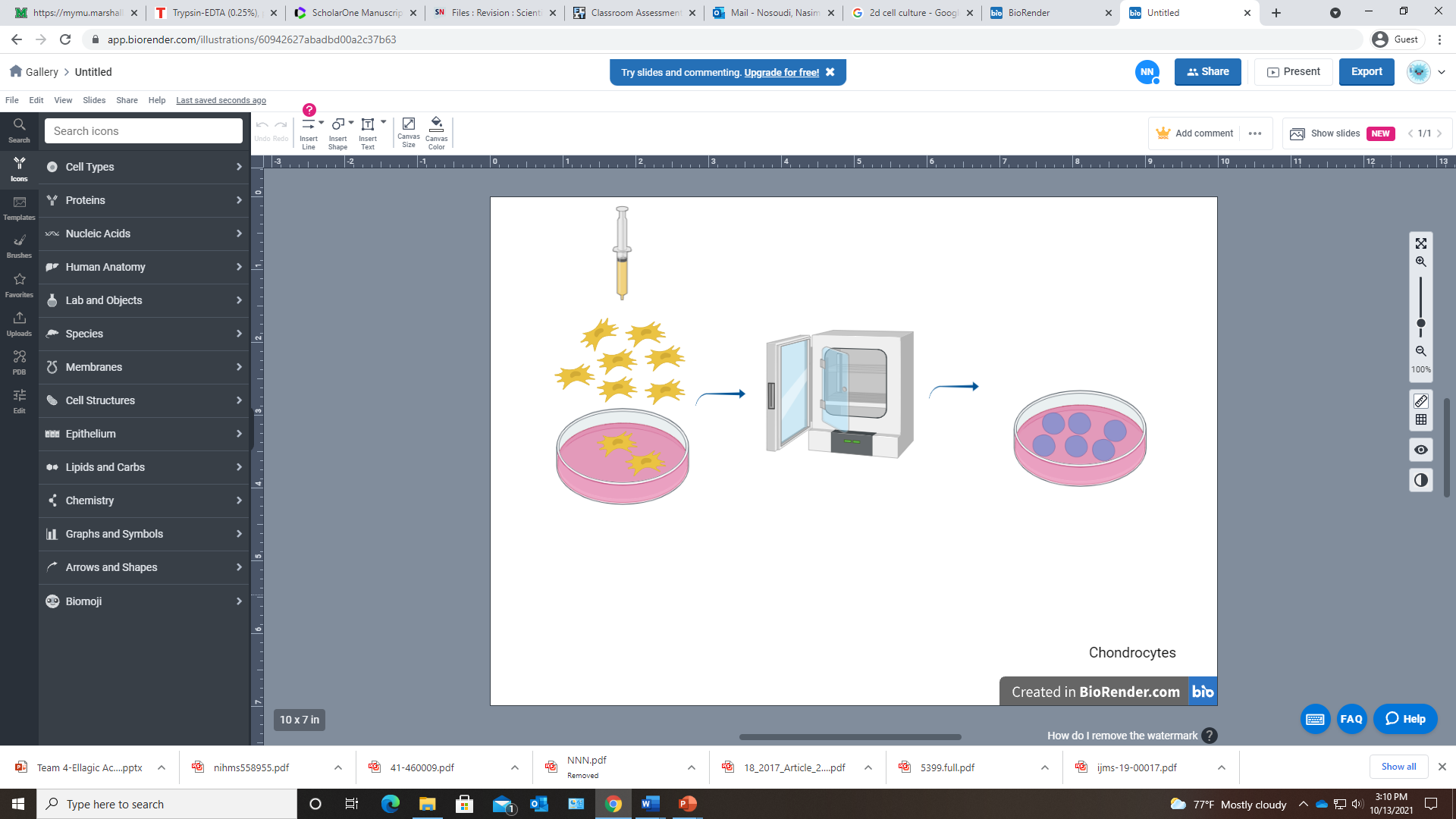
*

**Supplementary figure S9**: Graphical abstract
